# *Spiradicliskarstana* (Rubiaceae), a new species from Yunnan, China

**DOI:** 10.3897/phytokeys.117.28281

**Published:** 2019-02-04

**Authors:** Lei Wu, Xiong Li, Wen-Jian Liu, Quan-Ru Liu

**Affiliations:** 1 College of Forestry, Central South University of Forestry and Technology, Changsha 410004, Hunan, China Central South University of Forestry and Technology Changsha China; 2 College of Life Sciences, Beijing Normal University, Beijing 100875, Beijing, China Beijing Normal University Beijing China

**Keywords:** *
Spiradiclis
*, Rubiaceae, taxonomy, karst, China

## Abstract

*Spiradicliskarstana*, a new species of *Spiradiclis* (Rubiaceae) collected from Yunnan, China, is described for the first time. It is morphologically close to *S.jingxiensis*, but differs from the latter mainly by its inflorescences with 5–9 flowers, its 1.5–2.4 mm long peduncles, its stipules shorter than 1 mm and the 5–12 pairs of secondary veins. The conservation status is assessed as “Vulnerable” (VU) according to the IUCN Red List Categories and Criteria.

## Introduction

*Spiradiclis* Blume, a small genus of Rubiaceae as currently circumscribed (Lo 1998, 1999), consists of two subgenera and ca. 50 species. Most of them are widely scattered in southern and south-western China and north-western India, but a few occur in the Indo-China Peninsula (Robbrecht 1988, Deb and Rout 1989, Lo 1999, Chen and Taylor 2011, Wu et al. 2015a, b, 2016, Wang 2016, Pan et al. 2016, Liu et al. 2017). Most representatives of the genus are narrowly distributed in karst regions, generally accompanied by ferns and *Begonia* L., *Elatostema* J.R.Forst. & G.Forst. and Gesneriaceae. During flowering, species of *Spiradiclis* are sometimes confused with *Ophiorrhiza* L. (Wu et al. 2015a), but they can be distinguished from the latter and other related genera by the linear-oblong or subglobose capsules that dehisce with four valves when mature (Lo 1999, Wu et al. 2015b). In China, nearly 47 species have been found, of which ten species have been published in the last four years (Deng et al. 2014, Wu et al. 2015a, b, 2016, Wang et al. 2015, Wen et al. 2015, Wang 2016, Pan et al. 2016, Liu et al. 2017).

In 2017, a plant lover, Mr. Ming-Feng Long, found a population of *Spiradiclis* on the cliff of a karst hill in Malipo county, SE Yunnan and contacted the authors for the identification. The individuals were first identified as *Spiradiclisjingxiensis* R.J.Wang as they shared a similar habitat and morphological characters such as prostrate or decumbent habit, distylous and purple-reddish flowers with slender salverform corollas. After a further comparison of specimens, however, these individuals can be distinguished from *Spiradiclisjingxiensis* mainly by their puberulent to subglabrous stems, leaves, stipules, peduncles and calyces (vs. dense pubescence), their ovate-triangular stipules, shorter than 1 mm (vs. linear, 1.5–3.0 mm long), their elliptic to oblong leaf blades (vs. ovate to broadly ovate), their 5–12 pairs of secondary veins (vs. 4–5 pairs) and their inflorescences with 5–9 flowers (vs. 1–2 flowers). Therefore, the specimens are assumed to represent an undescribed new taxon, which is here described.

## Materials and methods

Most materials are deposited at the herbarium of forest plants in the Central South University of Forestry and Technology (**CSFI**), only one residing at Guangxi Institute of Botany, Guangxi Zhuang Autonomous Region and Chinese Academy of Sciences (**IBK**). Morphological observations of the new species have been carried out, based on field observations as well as on dry specimens. The conservation status of the new species is evaluated in accordance with IUCN guidelines (2016).

## Taxonomy

### Spiradiclis karstana

Taxon classificationPlantaeGentianalesRubiaceae

L.Wu, X.Li & Q.R.Liu sp. nov.

urn:lsid:ipni.org:names:77194418-1

Figures 1 , 2

#### Diagnosis.

Similar to *S.jingxiensis*, but differing from this species by the ovate-triangular stipules less than 1 mm long (vs. stipules linear, 1.5–3.0 mm long), the elliptic to oblong leaf blades (vs. ovate to broadly ovate), the 5–12 pairs of secondary veins (vs. 4–5 pairs) and the inflorescences with 5–9 flowers (vs. inflorescences with 1–2 flowers).

#### Type.

CHINA. Yunnan Province: Malipo County, Mali town, Luoshuidong village, 23°03'N, 104°43'E, 900 m alt., 11 Apr. 2018, *Ming-Feng Long & Lei Wu MLP0002* (holotype: CFSI; isotype: CFSI).

Perennial herbs, up to 8 cm in height, prostrate or decumbent, usually rooting at nodes; stems terete, basal part usually woody, densely puberulent (trichomes white). Petiole 2–7 mm long; leaf blade elliptic to oblong, 0.8–4.5 × 0.5–1.6 cm, drying papery, adaxially green, abaxially yellowish-green, adaxially puberulent to subglabrous, abaxially puberulent, densely pubescent along principal veins on lower surface; base cuneate and somewhat decurrent, apex obtuse to acute; secondary veins 5–12 pairs, both midrib and secondary veins adaxially impressed, abaxially prominent; stipules ovate-triangular or sometimes 2-lobed, 0.2–0.8 mm long, densely puberulent outside, usually deciduous. Inflorescences terminal, cymose, 5–9-flowered, puberulent; peduncles short, 1.5–2.4 mm long, puberulent; bracts subulate, 0.5–1.5 mm long, puberulent. Flowers distylous; pedicels 1.0–2.5 mm long, puberulent. Calyx puberulent to subglabrous outside; hypanthium obconic, 1.2–1.7 mm long; calyx lobes equal, lanceolate to narrowly triangular, 2–2.5 mm long, acute at apex. Corolla 5-merous, slenderly salverform, purple-reddish, with a deep purple-reddish ring around the throat, glabrous outside; tube 15–25 mm long; lobes broadly ovate, 3.5–6.0 × 3.0–5.0 mm, apically rostrate-incurved; stamens completely included in the corolla tube at anthesis in both flower morphs; ovary 2-celled. Long-styled flowers: inside with a ring of hairs near the stamens; stamens inserted near the base and stigma positioned a little below the throat of the corolla tube; anthers linear, ca. 2.0–2.4 mm long; stigma 2-lobed, lobes elliptic, ca. 1.2 mm long. Short-styled flowers: inside with sparse pubescence near the base of the corolla tube; stamens inserted above the middle and stigma positioned near the base of the corolla tube, anthers linear, ca. 2.5 mm; stigma 2-lobed, lobes ovate-lanceolate, ca. 1.5 mm long. Capsule obovoid, 2.5–3.5 mm long, subglabrous, opened at the top when mature. Seeds ca. 12 per capsule.

**Figure 1.**
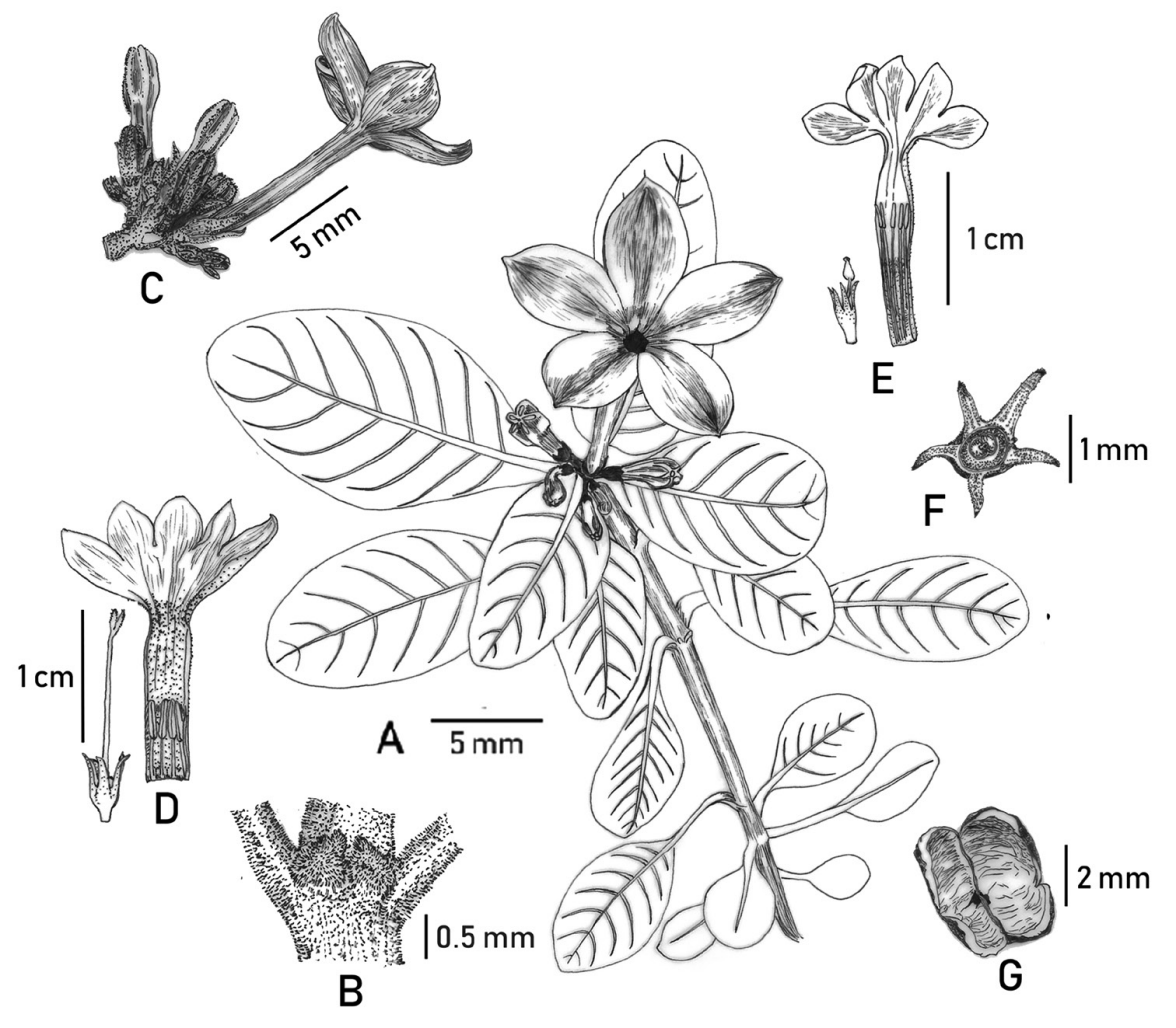
*Spiradicliskarstana* sp. nov. **A** habit **B** stipule **C** inflorescence, side view **D** long-styled flower **E** short-styled flower **F** calyx, frontal view, showing disc **G** remnant of dehiscent capsule, frontal view. Drawn by Xin-Yi Zeng.

**Figure 2.**
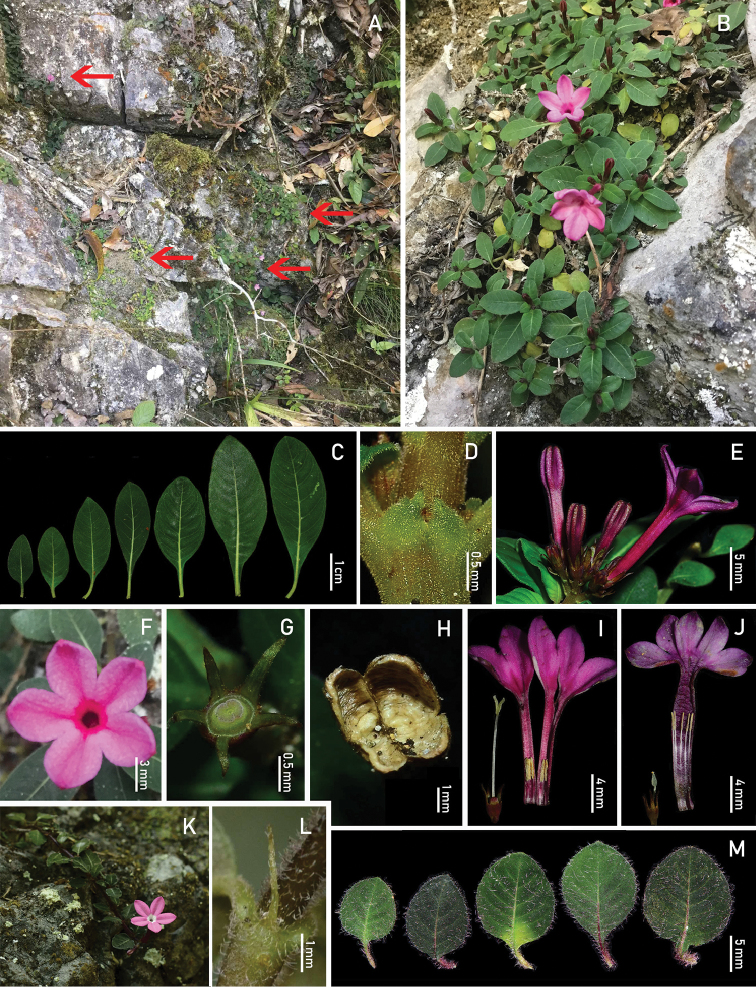
*Spiradicliskarstana* sp. nov. **A** habitat (The arrows show the places of growth) **B** habit **C** variation range of leaves **D** stipule **E** inflorescence, side view **F** long-styled flower, frontal view **G** disc and calyx **H** remnant of dehiscent capsule, frontal view **I** long-styled flower **J** short-styled flower. *Spiradiclisjingxiensis***K** habit **L** stipule **M** variation range of leaves. Photos by Lei Wu, Ming-Feng Long and Xin-Xin Zhu.

#### Phenology.

Flowers and fruits were observed in April. We think flowering and fruiting may extend till May, because many flowers in April were still in bud.

#### Distribution and habitat.

*Spiradicliskarstana* is known only from the crevices of forested cliffs at altitudes ranging from 800 to 1600 m in the karst area of SE Yunnan. This part of Yunnan is covered by evergreen rain forests that are highly similar to those in Indo-Malaysia (Zhu 2013) and are dominated by species from Magnoliaceae, Lauraceae, Dipterocarpaceae and Annonaceae.

#### Etymology.

The specific epithet refers to the habitat of the new species.

#### Specimens examined (paratypes).

**CHINA. Yunnan Province.** Malipo County, Mali town, Luoshuidong village, 25 Apr 2017, *Ming-Feng Long MLP 0001* (CFSI); Mengzhi city, Shuitian town, on a karst cliff, alt. 1650 m, 29 Apr 2017, *Meng-Qi Han et al. HMQ0001* (IBK).

#### Conservation status.

So far, only two populations with about 300 individuals each have been found, notably near the towns of Mali and of Shuitian. The area of occupancy is estimated to be less than 4 km^2^. Despite the fact that only two populations with relatively few individuals are known, the status of *Spiradicliskarstana* is not pessimistic. It may count on the strong adaptability of the species to the severe habitat where interference from humans is usually weak and where it is very hard for people to reach and the efficient government policy to protect the local vegetation (Lai et al. 2000, Sui and Chen 2006, Zhu et al. 2007). Considering all of the above, this species is therefore assigned a status of “Vulnerable” [VU B2ab(ii, iii, iv)] according to the IUCN criteria (2016).

## Discussion

Karst ecosystems are known to be distinctive in vegetation and biodiversity and have an extreme and exceptional habitat that can provide the opportunity for speciation and radiation (Myers et al. 2000, Zhu 2007, Biswas 2009). As mentioned above, most representatives of *Spiradiclis* prefer this unique ecosystem. It may be the reason why this small genus shows fairly complex and diverse habit and morphology. Until now, 11 known species of *Spiradiclis*, viz., *S.danxiashanensis* R.J.Wang, *S.elliptica* Y.M.Shui & W.H.Chen, *S.glandulosa* L.Wu & Q.R.Liu, *S.guangdongensis* H.S.Luo, *S.hainanensis* H.S.Luo, *S.jingxiensis* R.J.Wang, *S.longanensis* R.J.Wang, *S.pauciflora* L.Wu & Q.R.Liu, *S.pengshuiensis* B.Pan & R.J.Wang and *S.umbelliformis* H.S.Luo, as well as this new one, are prostrate or decumbent herbs without erect stems and usually with roots at the nodes (Sui and Chen 2006, Chen and Taylor 2011, Wu et al. 2015a, 2015b, Wang et al. 2015, Wen et al. 2015, Wang 2016, Pan et al. 2016). The following key is provided to the prostrate or decumbent *Spiradiclis* species worldwide.

### A key to the prostrate or decumbent species in *Spiradiclis*. This key was adapted from Chen and Taylor (2011)

**Table:** 

1	Corolla tubes shorter than 10 mm	**2**
–	Corolla tubes 10–25 mm	**5**
2	Leaf blades usually 2–10 cm long, second veins 7–9 pairs; corolla tubes 2–3 mm long	**3**
–	Leaf blades 0.5–2 cm long, second veins 3–4 pairs; corolla tubes longer than 7 mm	**4**
3	Stem densely hairy; bracts linear	*** S. longanensis ***
–	Stem subglabrous; bracts oblong	*** S. elliptica ***
4	Stipules shorter than 1 mm; inflorescences 3–7-flowered	*** S. pauciflora ***
–	Stipules 1.5–2.5 mm long; inflorescences 2–3-flowered	*** S. hainanensis ***
5	Stipules 0.3–1.2 mm long, caducous	**6**
–	Stipules 1.5–5 mm long, persistent	**7**
6	Stems and leaves densely pubescent; leaf blades ovate; calyx lobes 0.8–1.5 mm long	*** S. pengshuiensis ***
–	Stems and leaves puberulent; leaf blades elliptic to oblong; calyx lobes 2–2.5 mm long	*** S. karstana ***
7	Calyx lobes 0.6–1.5 mm long	**8**
–	Calyx lobes 2–6 mm long	**9**
8	Leaf blades 1.5–4 cm long; inflorescences 4–10-flowered	*** S. umbelliformis ***
–	Leaf blades 0.3–1.1 cm long; inflorescences 1–2-flowered	*** S. jingxiensis ***
9	Corolla glabrous outside, tubes 16–18 mm long	*** S. glandulosa ***
–	Corolla subglabrous outside, tubes 11–15 m long; stigmas excluded in long-styled flowers	**10**
10	Corollas salverform; peduncles 1.3–2.8 cm	*** S. danxiashanensis ***
–	Corollas slenderly funnelform; peduncles shorter than 1 cm	*** S. guangdongensis ***

## Supplementary Material

XML Treatment for Spiradiclis karstana
